# Archaeal DnaG contains a conserved N-terminal RNA-binding domain and enables tailing of rRNA by the exosome

**DOI:** 10.1093/nar/gku969

**Published:** 2014-10-17

**Authors:** Linlin Hou, Gabriele Klug, Elena Evguenieva-Hackenberg

**Affiliations:** Institute of Microbiology and Molecular Biology, Heinrich-Buff-Ring 26–32, D-35392 Gießen, Germany

## Abstract

The archaeal exosome is a phosphorolytic 3′–5′ exoribonuclease complex. In a reverse reaction it synthesizes A-rich RNA tails. Its RNA-binding cap comprises the eukaryotic orthologs Rrp4 and Csl4, and an archaea-specific subunit annotated as DnaG. In *Sulfolobus solfataricus* DnaG and Rrp4 but not Csl4 show preference for poly(rA). Archaeal DnaG contains N- and C-terminal domains (NTD and CTD) of unknown function flanking a TOPRIM domain. We found that the NT and TOPRIM domains have comparable, high conservation in all archaea, while the CTD conservation correlates with the presence of exosome. We show that the NTD is a novel RNA-binding domain with poly(rA)-preference cooperating with the TOPRIM domain in binding of RNA. Consistently, a fusion protein containing full-length Csl4 and NTD of DnaG led to enhanced degradation of A-rich RNA by the exosome. We also found that DnaG strongly binds native and *in* *vitro* transcribed rRNA and enables its polynucleotidylation by the exosome. Furthermore, rRNA-derived transcripts with heteropolymeric tails were degraded faster by the exosome than their non-tailed variants. Based on our data, we propose that archaeal DnaG is an RNA-binding protein, which, in the context of the exosome, is involved in targeting of stable RNA for degradation.

## INTRODUCTION

The RNA degrading exosome is a protein complex found in eukarya and archaea (1–3). It is composed of a structurally conserved nine-subunit core, which also shows similarities to bacterial polynucleotide phosphorylase (PNPase), and contains additional subunits (4–9). The nine-subunit core of the eukaryotic exosome is essential but catalytically inactive and additional eukarya-specific subunits are responsible for the ribonucleolytic activity (9–11). In contrast, the archaeal nine-subunit exosome is a 3′–5′-exoribonuclease like PNPase (4,8) and strongly interacts with a protein annotated as DnaG (3,8,12,13). The archaeal exosome and bacterial PNPase have not only structural but also functional similarities — they degrade RNA phosphorolytically using inorganic phosphate and producing rNDPs, and in a reverse reaction they synthesize heteropolymeric RNA tails (8,14–16). It was suggested that the heteropolymeric RNA tails found in prokaryotes destabilize RNA enabling efficient binding of 3′–5′ exoribonucleases including PNPase or exosome (15,17). Such destabilization mechanism is known for short poly(A)-tails synthesized by poly(A)-polymerase in enterobacteria (18,19) and by non-canonical poly(A)-polymerases in eukaryotes, where the polyadenylation of rRNA precursors is a prerequisite for their degradation by the eukaryotic exosome (20,21).

**Figure 1.**
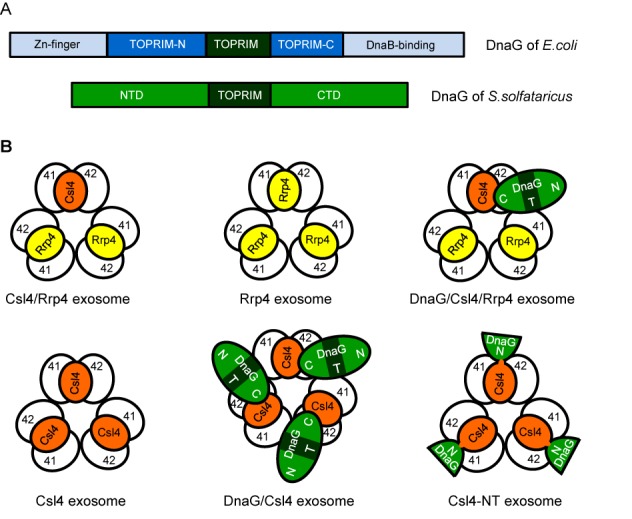
Comparison of bacterial and archaeal DnaG and composition of reconstituted *Sulfolobus solfataricus* exosomes. (**A**) Domain composition of DnaG from *Escherichia coli* and *S. solfataricus*. Toprim-N and Toprim-C are the N-and C-terminal parts of the crystallized part of *E. coli* DnaG (40), which do not show similarity to archaeal DnaG proteins. NTD, N-terminal domain; CTD, C-terminal domain. (**B**) Schematic illustration of different exosomal complexes which were reconstituted previously and/or in this work. 41, Rrp41; 42, Rrp42; N, T and C, NTD, TOPRIM domain and CTD of DnaG. The Toprim domain is in dark green. Top views based on crystal structures of the Rrp41/Rrp42 hexamer (4), nine-subunit exosomes with homotrimeric, Rrp4 or Csl4 containing caps (5,22) and biochemical data for DnaG-containing exosomes (33).The Csl4-NT-exosome contains a homotrimeric cap build of the fusion protein Csl4-NT, which comprises full-length Csl4 and the NTD of DnaG.

While the structure and function of the archaeal nine-subunit exosome is well understood (4,5,22–24), little is known about the role of archaeal DnaG in the context of the exosome. Its annotation is based on its central topoisomerase/primase (TOPRIM) domain (25,26) and nothing is known about the function of its N-terminal and C-terminal domains (NTD and CTD, respectively, Figure 1A). The archaeal nine-subunit exosome is formed by orthologs of the eukaryotic exosomal subunits Rrp41, Rrp42, Rrp4 and Csl4. The RNase PH-domain containing subunits Rrp41 and Rrp42 are arranged in a catalytically active hexamer, on the top of which a trimeric cap composed of the RNA-binding proteins Rrp4 and Csl4 is bound (Figure 1B; 4,5,22–24). The RNA-binding cap increases the efficiency of degradation of poly(A) and heteropolymeric RNA by the recombinant archaeal exosome (8,27–30). While *in vivo* the exosome contains both Rrp4 and Csl4 (31), *in vitro* complexes with homotrimeric, Rrp4 or Csl4 containing caps (Rrp4 exosome or Csl4 exosome) can be reconstituted (Figure 1B; 5,8). Their comparative analysis revealed that Rrp4 confers poly(A)-preference to the exosome of the hyperthermophilic and acidophilic archaeon *Sulfolobus solfataricus* (32), while Csl4 is needed for the interaction of the complex with DnaG (33; Figure 1B). Furthermore it was shown that DnaG preferentially binds poly(A) RNA in electrophoretic mobility shift assay (EMSA) and increases the poly(A)-preference of the *S. solfataricus* exosome even in the presence of Rrp4 (33). This suggested that DnaG is a part of the RNA-binding platform of the *S. solfataricus* exosome and modulates its substrate specificity (33). However, it remained unknown how DnaG interacts with the exosome and with RNA substrates.

**Figure 2.**
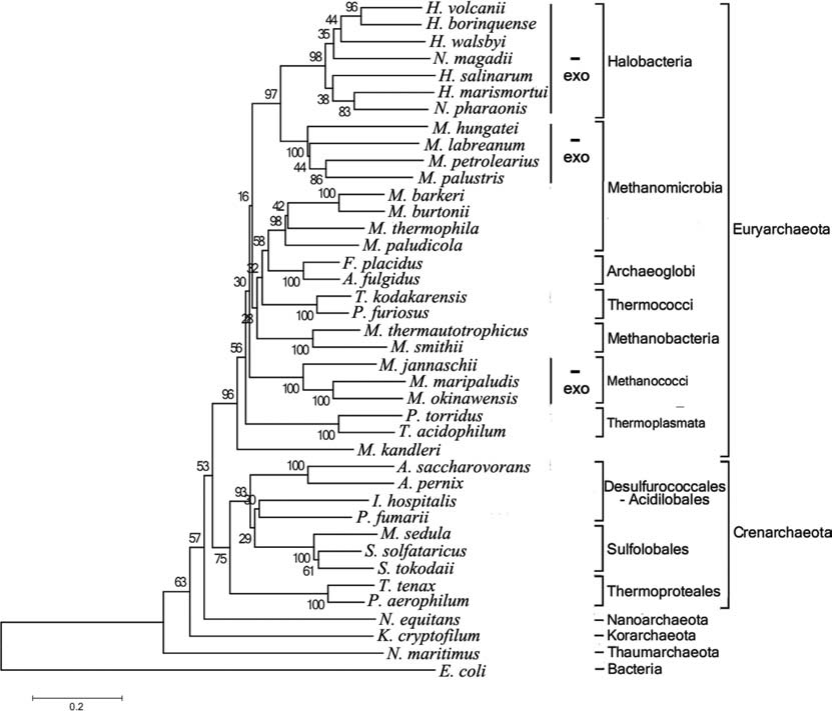
Phylogenetic analysis of DnaG proteins in Archaea. Genes encoding DnaG homologs exist in all genome sequenced archaea. The neighbor-joining phylogenetic tree of DnaG proteins is based on full-length protein sequences obtained from NCBI (http://www.ncbi.nlm.nih.gov/). Archaea which do not harbor genes for the core exosomal subunits Rrp41 and Rrp42 are marked with ‘-exo’. In Methanomicrobia, exosome-less and exosome-containing species group separately. *H. volcanii, Haloferax volcanii; H. borinquense, Halogeometricum borinquense; H. walsbyi, Haloquadratum walsbyi; N. magadii, Natrialba magadii; H. salinarum, Halobacterium salinarum; H. marismortui, Haloarcula marismortui; N. pharaonis, Natronomonas pharaonis; M. hungatei, Methanospirillum hungatei; M. labreanum, Methanocorpusculum labreanum; M. petrolearius, Methanoplanus petrolearius; M. palustris, Methanosphaerula palustris; M. barkeri, Methanosarcina barkeri; M. burtonii, Methanococcoides burtonii; M. thermophila, Methanosaeta thermophila; M. paludicola, Methanocella paludicola; F. placidus, Ferroglobus placidus; A. fulgidus, Archaeoglobus fulgidus; T. kodakarensis, Thermococcus kodakarensis; P. furiosus, Pyrococcus furiosus; M. thermautotrophicus, Methanothermobacter thermautotrophicus; M. smithii, Methanobrevibacter smithii; M. jannaschii, Methanocaldococcus jannaschii; M. maripaludis, Methanococcus maripaludis; M. okinawensis, Methanothermococcus okinawensis; P. torridus, Picrophilus torridus; T. acidophilum, Thermoplasma acidophilum; M. kandleri, Methanopyrus kandleri; A. saccharovorans, Acidilobus saccharovorans; A. pernix, Aeropyrum pernix; I. hospitalis, Ignicoccus hospitalis; P. fumarii, Pyrolobus fumarii; M. sedula, Metallosphaera sedula; S. solfataricus, Sulfolobus solfataricus; S. tokodaii, Sulfolobus tokodaii; T. tenax, Thermoproteus tenax; P. aerophilum, Pyrobaculum aerophilum; N. equitans, Nanoarchaeum equitans; K. cryptofilum, Candidatus Korarchaeum cryptofilum; N. maritimus, Nitrosopumilus maritimus; E. coli, Escherichia coli.*

The tight interaction between archaeal exosome and DnaG was documented for several archaeal species (3,8,12,13), and fractionation of cell-free extracts followed by co-immunoprecipitation (CoIP) strongly suggested that in *S. solfataricus* DnaG is an indispensable part of the exosome (31). On the other hand, DnaG is ubiquitous in all genome-sequenced archaea, while the exosome is missing in *Methanococci*, *Halobacteria* and some *Methanomicrobia* (Figure 2 and Supplementary Figure S1; ref. 2,15,34). The high conservation of DnaG in archaea can be explained by the assumption that it plays an important role in RNA metabolism even in the absence of exosome, and/or by its putative role as a primase, in accordance to its annotation and recent biochemical data (35,36). The primase synthesizes *de novo* short RNA primers during chromosome replication. (37). Archaea possess a two-subunit primase PriS/PriL of eukaryotic type, which was characterized *in vitro* (38,39). This primase shows strong interactions with components of the archaeal replication network in pull-down assays with *Thermococcus kodakarensis* cell-free extracts, while the putative bacterial-type primase DnaG interacts with the exosome instead (13). However, it was published that DnaG of *S. solfataricus* exhibits primase activity *in vitro*, and this activity is decreased by mutations of conserved residues in the TOPRIM domain. Furthermore, an interaction was detected between *S. solfataricus* DnaG and the archaeal minichromosome maintenance (MCM) helicase in yeast two-hybrid system and *in vitro* pull-down assays. Based on this, it was suggested that archaeal DnaG may have a dual function in the cell, as a part of the exosome and as a bacterial-type primase (35,36).

The bacterial primase DnaG is composed of an NTD containing a Zn-finger motif involved in DNA binding, the central, catalytic TOPRIM domain and a CTD necessary for the interaction with the replicative helicase DnaB (Figure 1A, refs. 26,37,40,41). Assuming that a primase needs a DNA-binding domain while a protein important for RNA metabolism should possess an RNA-binding domain, we decided to characterize the NTD and CTD of *S. solfataricus* DnaG. We found that the NTD is a conserved archaeal RNA-binding domain cooperating with the TOPRIM domain in binding of RNA substrates, while the CTD is important for the binding to the exosome. Furthermore, we show that *in vitro* the exosome needs DnaG for post-transcriptional tailing of native rRNA, and that heteropolymeric tails enhance the degradation of rRNA transcripts. Our data strongly suggest that DnaG is a conserved archaeal RNA-binding protein, which participates in the degradation of stable RNAs in *S. solfataricus*.

## MATERIALS AND METHODS

### Phylogenetic analysis of archaeal DnaG proteins

Sequences of DnaG proteins were obtained from NCBI (http://www.ncbi.nlm.nih.gov/) and aligned using Clustal X 2.1 (http://www.clustal.org/clustal2/). The neighbor-joining phylogenetic tree of DnaG proteins was generated by using MEGA 5.2 with 500 bootstrap replicates (MEGA 5.2 http://www.megasoftware.net/). The Poisson correction method was used to compute the evolutionary distances which are in the units of the number of amino acid substitutions per site.

### Preparation of wild-type and mutant proteins

Recombinant hexahistidine-tagged DnaG, Rrp4, Csl4, Rrp41 and Rrp42, and streptavidin-tagged (Strep-tagged) Csl4 were expressed and purified as previously described (33). DnaG-E175Q was kindly provided by Dr. Michael A. Trakselis (Pittsburgh, USA) and purified as previously described (35). Primers used for the construction of mutant proteins are shown in Supplementary Table S1. DnaG-K6AY7A, DnaG-K6A and DnaG-Y7A genes were generated by standard overlap extension polymerase chain reaction (PCR) (42) and cloned into pET15b vector using NcoI and NdeI restriction sites. Overlap extension PCR was also used for the fusion of DNA encoding the N-terminal 156 amino acid residues of DnaG to the 3′-end of the Csl4 gene. The PCR product was cloned into pET15b vector using NdeI and BamHI restriction sites. Both constructs were expressed in *Escherichia coli* BL21-Goldenplus (DE3). Cells producing DnaG-K6AY7A, DnaG-K6A or DnaG-Y7A were sonicated in buffer containing 50 mM HEPES (pH 8.0), 100 mM NaCl and 10 mM β-mercaptoethanol, 10% glycerol. The cell-free extract was heated at 75°C for 20 min and the soluble protein was purified through HiTrap HP Q and HiLoad^®^ 26/60 Superdex^®^ 200 PG columns. DnaG-ΔNT was purified using the same lysis buffer, heat treatment and Ni-NTA resin. Cells producing the fusion protein Csl4-DnaG′ named Csl4-NT were sonicated in buffer containing 50 mM HEPES (pH 8.0) and 1 M NaCl. After incubation at 75°C for 20 min, the soluble protein was purified using Ni-NTA resin.

### Protein–protein interaction studies

Interactions between the Csl4 exosome and His_6_-DnaG-ΔCT or DnaG-K6AY7A were analyzed by pull-down assays, in which reconstituted Csl4 exosome and cell-free extracts of *E. coli* expressing one of the DnaG variants were used (33). The Csl4 exosome was reconstituted by mixing His_6_-Rrp41 and His_6_-Rrp42 with Strep-tagged Csl4 (0.8 mg of each protein) in a final volume of 5 ml in buffer P0 (10 mM Tris (pH 8.0), 5 mM MgCl_2_, 0.5 mM ethylenediaminetetraacetic acid (EDTA), 200 mM NaCl, 5% glycerol, 0.05% Tween 20 and 0.2 mM dithiotreitol (DTT)) and incubating at room temperature for 2 h. After treatment at 75°C for 10 min and centrifugation at 13 000 *g* for 10 min, the supernatant containing reconstituted Csl4 exosome was collected. To prepare cell-free extracts of *E. coli*, 1 l culture expressing His_6_-DnaG-ΔCT or DnaG-K6AY7A was harvested at OD_600_ = 0.6 after 3 h of induction with 1 mM isopropyl-beta-D-thiogalactopyranoside (IPTG). The cell pellet was resuspended in 20 ml of buffer containing 50 mM HEPES (pH 8.0), 100 mM NaCl and 10 mM β-mercaptoethanol, 10% glycerol. After sonication and centrifugation at 13 000 *g* for 20 min, 5 ml of the supernatant was mixed with the Csl4 exosome. The mixture was incubated in buffer P0 for 2 h at room temperature. Then it was passed twice through a column with 1 ml *Strep*-Tactin^®^ Sepharose^®^. The *Strep*-Tactin Sepharose was washed with buffer P0 and eluted with 200 μl buffer containing 100 mM Tris (pH 8.0), 150 mM NaCl, 1 mM EDTA and 5 mM D-desthiobiotin.

Interaction between the Csl4 exosome and His_6_-DnaG-ΔNT was analyzed by CoIP assay using Rrp41-directed serum as previously described (8,31,33). All proteins in this assay were His-tagged. Protein fractions were analyzed by sodium dodecylsulphate-polyacrylamide gel electrophoresis (SDS-PAGE) and silver-staining. For western blot analysis, protein samples were separated in 12% SDS-polyacrylamide (PAA) gel and then transferred to Protran nitrocellulose membrane (Whatman). Western blot analysis was performed as described (31).

### Circular dichroism spectroscopy

Circular dichroism (CD) spectra were recorded in a JASCO J-710 circular dichroism spectrophotometer at ambient temperature. DnaG (2.17 μM) and the variant DnaG-K6AY7A (2.21 μM) were measured in a cell with 0.05 cm path in 10 mM Na_2_HPO_4_-NaH_2_PO_4_ (pH 8.0), 50 mM NaCl_2_.

### Preparation of RNA substrates

Generation and purification of 5′-labeled poly(rA)_30_ and the following internally labeled or unlabeled *in vitro* transcripts was previously described (30,33): (i) native tail RNA of 59 nt (corresponding to an RNA tail detected in *S. solfataricus*), (ii) MCS-RNA of 30 nt (corresponding to a part of a multiple cloning site of a plasmid) and (iii) 3′-end 16S rRNA transcript of 163 nt. Native 5S rRNA was purified and labeled as follows. Total RNA was isolated using TRIzol, separated on 10% polyacrylamide-urea gel and stained with ethidium bromide. The gel slice containing 5S rRNA was cut out, and RNA was eluted overnight in buffer composed of 500 mM NaOAc (pH 5.2), 1 mM EDTA and 2.5% phenol/chloroform. After phenol-chloroform extraction and ethanol precipitation, 5S rRNA was labeled at the 5′-end using [α-^32^P] ATP. For the generation of internally labeled 5S rRNA (sequence according to the Comparative RNA Web Site and Project, http://www.rna.ccbb.utexas.edu, ref. 43), the 5S rRNA gene was amplified with the primers indicated in Supplementary Table S1 and *in vitro* transcription in presence of [α-^32^P] ribonucleoside uridine triphosphate (rUTP) was performed as described (30,33). The sequence of the 20 nt heteropolymeric tail added at the 3′-end of the 3′ 16S rRNA and 5S rRNA transcripts is AAAGGGGGAUAAAAUAAAGA and corresponds to a tail previously detected in *S. solfataricus* (15).

### RNA degradation and polyadenylation assays

Degradation and polyadenylation assays were carried out with 1.000 counts per minute (c.p.m.) of radioactively labeled substrate in a 10 μl reaction mixture containing 20 mM HEPES (pH 7.9), 60 mM KCl, 8 mM MgCl_2_, 0.1 mM EDTA, 2 mM DTT and 10 mM K_2_HPO_4_ (degradation assays) or 10 mM ribonucleoside adenine diphosphate (rADP) (polyadenylation assays). In each assay, 0.03 pmol/μl of a reconstituted complex was used. The concentration of substrate in the assays is indicated in the figure legends. For the assays, Csl4 exosome, DnaG/Csl4 exosome and Csl4-NT exosome were reconstituted using His_6_-Csl4, DnaG-His_6_ or His_6_-Csl4-NT and equimolar amounts of thawed His_6_-Rrp41/His_6_-Rrp42 hexamer. The hexamer was prepared in buffer containing 10 mM Tris–HCl, pH 7.6 and 150 mM NaCl, heat treated at 75°C for 20 min, purified through gel filtration and stored at −80°C in aliquots (33). Repeated thawing was avoided. Rrp4/Csl4 exosome and DnaG/Rrp4/Csl4 exosome were reconstituted using Strep-tagged Csl4 and were purified by tandem chromatography using *Strep*-Tactin and Ni-NTA-Agarose as described (33). Enzymatic reactions were carried out at 60°C for the indicated time (min). Samples were analyzed in 16 or 10% denaturing PAA gels at 400 V and visualized by phosphorimaging. Signals were detected and quantified using a Bio-Rad molecular imager and Quantity One (Bio-Rad). For graphical representation, the radioactivity per lane was set to 100% and % remaining substrate was calculated.

### RNA-binding assays

Binding assays were carried out at room temperature for 5 min in a 10 μl reaction mixture containing 20 mM HEPES (pH 7.9), 60 mM KCl, 10 mM MgCl_2_, 10% glycerine, 2 mM DTT and 0.1 mM EDTA with the indicated amounts of proteins and RNA substrates. The reaction samples were resolved in 5% native PAA gels at 200 V and 4°C, and were visualized by phosphorimaging using a Bio-Rad molecular imager and Quantity One (Bio-Rad) (5,33).

## RESULTS

### High conservation of the N-terminal and TOPRIM domains is independent of the exosome

Genes encoding archaeal DnaG proteins are found in all genome-sequenced archaea regardless of presence or absence of an exosome (2,44). To learn more about the evolution of the archaeal DnaG proteins, we created a phylogentic tree based on the sequences of DnaG proteins from 39 representative archaeal species (Figure 2) and compared this tree to the 16S rRNA-based phylogenetic tree of 120 genome-sequenced archaea (Supplementary Figure S1). Both trees are congruent in the delineation of the phyla Euryarchaeota, Crenarchaeota, Nanoarchaota, Korarchaeota and Thaumarchaeota. Interestingly, the absence of exosome leads to major differences in the DnaG subtree of Euryarchaeota, which comprise exosome-containing and exosome-less representatives, when compared to the 16S rRNA tree. An informative example are *Methanomicrobia*. In the DnaG tree, exosome-less *Methanomicrobia* form a well-delineated cluster together with the exosome-less *Halobacteria*, while exosome-containing *Methanomicrobia* cluster together with *Archaeoglobi* and other exosome-containing archaea (Figure 2). This is in contrast to the 16S rRNA tree, where *Methanomicrobia* and *Halobacteria* are in a cluster well separated from *Archaeoglobi* and other Euryarchaeota (Supplementary Figure S1). *Methanococci*, which accordingly to the 16S rRNA tree are distantly related to *Methanomicrobia* and *Halobacteria*, also do not have an exosome. This may explain other differences between the DnaG- and 16S rRNA-based subtrees of Euryarchaeota (compare Figure 2 to Supplementary Figure S1).

**Figure 3.**
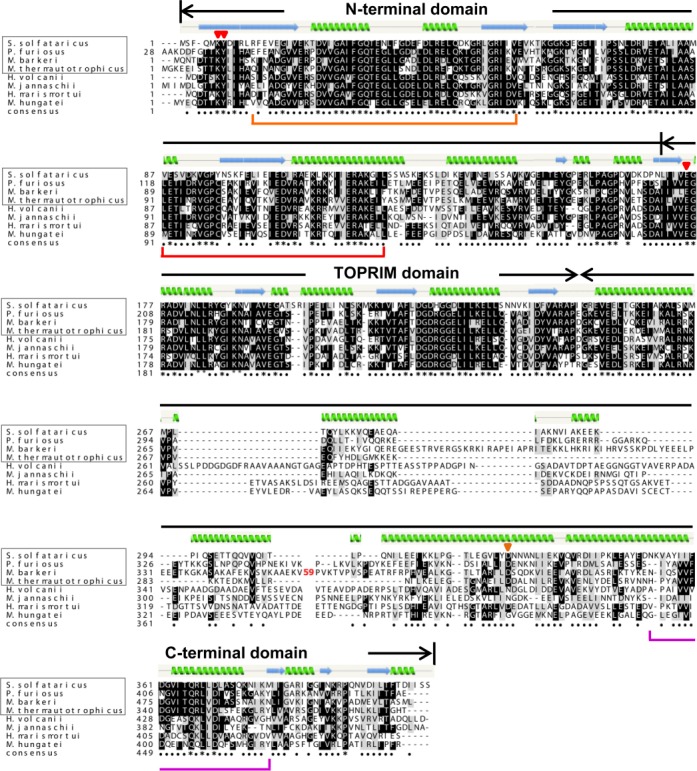
Multiple sequence alignment of DnaG using Clustal X-2.1. The secondary structure of *Sulfolobus solfataricus* DnaG was modeled with Phyre2 (http://www.sbg.bio.ic.ac.uk/phyre2/html). The domains of DnaG are marked above the alignment. Mutated residues in the N-terminal (K6 and Y7, this work) and TOPRIM (E175, (35)) domains of *S. solfataricus* DnaG are marked with red triangles above the alingment. In the C-terminal domain, the *S. solfataricus* D329 residue conserved in exosome-containing archaea is marked with an orange triangle above the alignment. 59 aa of the C-terminal domain of *Methanosarcina barkeri* were omitted from the analysis (marked with red 59 in the *M. barkeri* sequence). DnaG regions showing similarities to other proteins in bacteria and/or eukarya are marked below the alignment: orange line, similarity to bacterial RNA helicase; red line, similarity to mammalian ribosomal protein L32; purple line, similarity to transcription elongation factor Spt4/5 (compare to Supplementary Table S2). The archaeal species framed in rectangle are exosome-containing. The archaeal species out of the rectangle are exosome-less. *S. solfataricus, Sulfolobus solfataricus; P. furiosus, Pyrococcus furiosus; M. barkeri, Methanosarcina barkeri; M. thermautotrophicus, Methanothermobacter thermautotrophicus; H. volcanii, Haloferax volcanii; M. jannaschii, Methanocaldococcus jannaschii; H. marismortui, Haloarcula marismortui; M. hungatei, Methanospirillum hungatei.*

To get insight into similarities and differences between the individual domains of DnaG in different archaea, multiple alignment of eight DnaG sequences from species with and without exosome was performed (Figure 3). We found that the conservation of the NTD of DnaG is very high and is comparable to that of the TOPRIM domain. The CTD is less conserved and the conservation is even lower in exosome-less archaea. Additional alignments were performed with 14 DnaG sequences from exosome-less archaea only (Supplementary Figure S2) and with 16 DnaG sequences from exosome-containing archaea only (Supplementary Figure S3). These alignments confirmed the highly conserved nature of the NTD and the TOPRIM domain, and the lower conservation of the CTD, especially in exosome-less archaea. Three invariant residues were found in the CTD of exosome-less archaea, but it should be taken into account that in this case only sequences from Euryarchaeota were compared (Supplementary Figure S2). These residues are also present in DnaG from the exosome-containing Euryarchaeota and the Crenarchaeon *S. solfataricus* shown in Figure 3. In the last 100 aa of the CTD of exosome-containing archaea belonging to all five archaeal phyla, an invariant aspartate residue (D329 in *S. solfataricus*) and a cluster of conserved residues (F360 to D367 in *S. solfataricus*) were detected. This cluster is present in all analyzed exosome-containing species but *Nanoarchaeum equitans* (Supplementary Figure S3). The data suggest that in exosome-containing archaea with exception of *N. equitans*, the CTD of DnaG is involved in the interaction with the exosome.

We also searched for similarities between the NTD and CTD of archaeal DnaG and other proteins using Phyre2 (http://www.sbg.bio.ic.ac.uk/phyre2). The analysis was performed with DnaG from the exosome-containing *S. solfataricus* and the exosome-less *Methanocaldococcus jannaschii* (Supplementary Table S2). This analysis revealed that the NTD of DnaG from both archaeal species harbors a region with similarity to bacterial RNA helicases (in agreement with ref. 2) and another region with similarity to mammalian ribosomal protein L32. The most conserved region of the CTD of both species shows similarity to the transcription elongation factor Spt4/5 interacting with RNA polymerase (Supplementary Table S2, Figure 3, ref. 45). For essentially the same region of the CTD of *S. solfataricus* similarity to Rossmann fold was found (Supplementary Table S2).

### The CTD of DnaG is important for the interaction with the exosome

**Figure 4.**
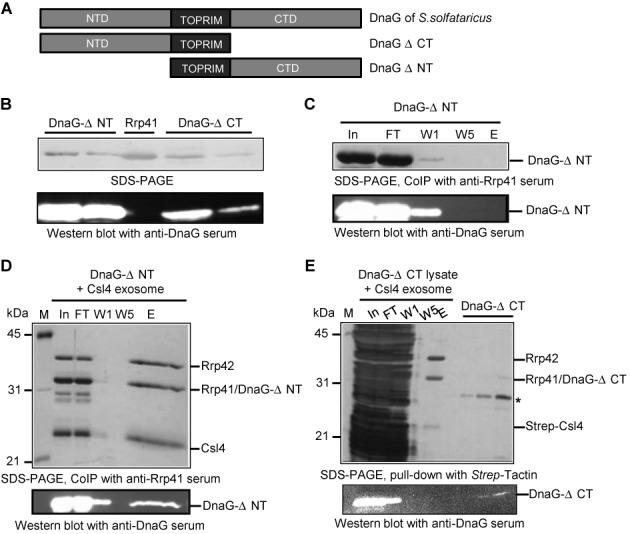
The CTD of DnaG is involved in the interaction with the Csl4 exosome. (**A**) Schematics of the used DnaG variants. (**B**)-(**E**), Upper panels show silver stained 12% SDS-PAA gels (SDS-PAGE) and lower panels show corresponding western blot analyses with polyclonal DnaG-directed antibodies. All recombinant proteins carry hexahistidine tags. The only exception is the Strep-tagged Csl4 in (**E**). Relevant proteins are marked on the right side of the panels. The size of marker proteins in kDa is given on the left side. (**B**) The DnaG-directed antibodies detect DnaG-ΔNT and DnaG-ΔCT but not Rrp41. The loaded, purified recombinant proteins are indicated above the upper panel. (**C**), (**D**) and (**E**) Results from coimmunoprecipitation experiments (CoIP) with polyclonal, Rrp41-directed antibodies or from a pull-down experiment with *Strep*-Tactin as indicated below the upper panels. In, input, the mixture of proteins used; FT, flow-through; W1, W5, the first and the last washing fractions; E, the elution fraction. (**C**) DnaG-ΔNT does not interact with the immobilized Rrp41-specific antibodies used for CoIP. Purified DnaG-ΔNT was used in the input fraction. (**D**) DnaG-ΔNT interacts with the Csl4 exosome. DnaG-ΔNT was mixed with reconstituted Csl4 exosome and subjected to CoIP. (**E**) No detectable interaction between DnaG-ΔCT and the Csl4 exosome. Cell-free extract of the *Escherichia coli* strain producing DnaG-ΔCT (DnaG-ΔCT lysate) was mixed with the reconstituted Strep-Csl4 exosome, and CoIP was performed. Dilutions of purified DnaG-ΔCT were loaded left to the elution fraction. The asterisk indicates an *E. coli* protein band which co-purifies with DnaG-ΔNT and DnaG-ΔCT.

To test experimentally which of the DnaG domains is responsible for the binding to the exosome, DnaG variants lacking either the NTD (His_6_-DnaG-ΔNT) or the CTD (His_6_-DnaG-ΔCT; see Figure 4A) were generated and used in protein–protein interaction assays with the exosome containing a homotrimeric Csl4 cap (Csl4 exosome). Since both truncated DnaG variants have the same length like Rrp41, it was necessary to discriminate them from His_6_-Rrp41 by western blot analysis with DnaG-specific serum. Figure 4B shows that both truncated His_6_-tagged DnaG variants but not His_6_-Rrp41 were detected using the DnaG-specific serum. Furthermore we noticed that the serum shows stronger signals for His_6_-DnaG-ΔNT than for His_6_-DnaG-ΔCT. We conclude that the specificity of the DnaG-directed serum is sufficient for our analysis.

Interaction between His_6_-DnaG-ΔNT and the Csl4 exosome was analyzed by CoIP with Rrp41-specific antibodies coupled to protein A-Sepharose beads. Previously we have shown that binding of full-length DnaG to the Csl4 exosome is easily detectable with this assay (33). Since all proteins used carry a His_6_-tag and the polyclonal antibodies were raised against His_6_-Rrp41, we performed a control immunoprecipitation experiment with His_6_-DnaG-ΔNT only. Figure 4C shows that His_6_-DnaG-ΔNT did not interact with the antibodies. Next His_6_-DnaG-ΔNT and the Csl4 exosome were mixed and CoIP was performed. SDS-PAGE and western blot with the anti-DnaG serum revealed that His_6_-DnaG-ΔNT was not present in the last washing fraction but was well detectable in the elution fraction (Figure 4D). We conclude that His_6_-DnaG-ΔNT interacts with the exosome.

Since the purified His_6_-DnaG-ΔCT protein shown in Figure 4B was highly unstable, a cell-free extract of the *E. coli* strain, in which the protein was produced, was directly used for interaction tests. The extract was mixed with the Csl4 exosome containing a Strep-tagged variant of Csl4. All other recombinant proteins were His_6_-tagged. Exosomal complexes were purified with *Strep*-Tactin Sepharose beads, and SDS-PAGE and western blot analysis with DnaG-specific antibodies were performed. His_6_-DnaG-ΔCT was well detectable in the input, flowthrough and the first washing fraction but was not detected in the elution fraction (Figure 4E). Since interaction between the Strep-tagged Csl4 exosome and full-length DnaG in *E. coli* cell-free extract was easily detectable by pull-down assays with *Strep*-Tactin Sepharose beads (for an example see Figure 7A below), we conclude that the CTD of DnaG is important for the binding to the archaeal exosome.

**Figure 5.**
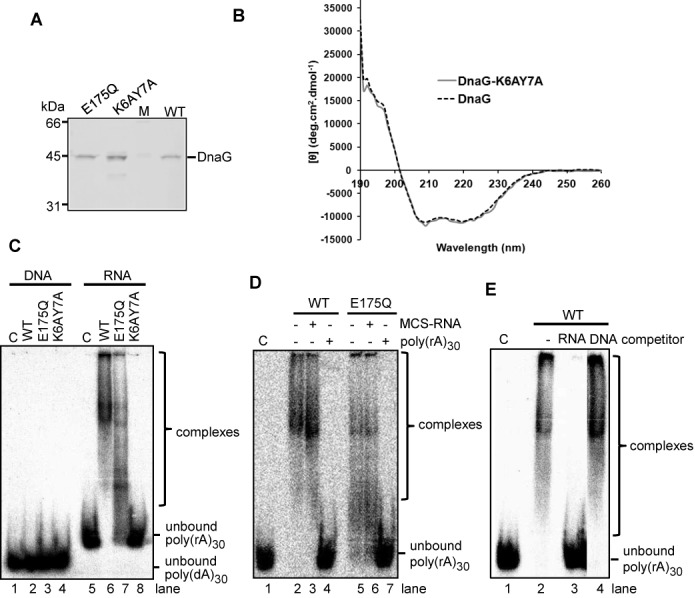
Conserved residues in the NTD are essential for the RNA binding activity of DnaG. (**A**) Coomassie stained SDS-PAA gel with purified wild-type DnaG (WT), mutated DnaG-E175Q with an amino acid exchange in the TOPRIM domain (E175Q) and mutated DnaG-K6AY7A protein with amino acid exchange in the NTD (K6AY7A). M, marker proteins, sizes in kDa are marked. (**B**) Circular dichroism analysis of WT DnaG and DnaG-K6AY7A at room temperature shows no change in the secondary structure elements of the mutant in comparison to the wild-type. (**C**), (**D**) and (**E**) show phosphorimages of EMSA in native 5% PAA gels with 25-fmol-labeled substrate and 2.5 pmol of WT DnaG or mutant proteins (marked above the panels). C, negative control without protein. Detected unbound substrates or complexes are marked on the right site of the panels. (C) EMSA with labeled poly(dA)_30_ (DNA) or poly(rA)_30_ (RNA). (D) EMSA with labeled poly(rA)_30_ and 40 pmol of unlabeled RNA (30-nt MCS-RNA or poly(rA)_30_) as competitor. The presence or absence of competitor is indicated above the panel. (E) EMSA with labeled poly(rA)_30_ and 2.5 pmol unlabeled poly(rA)_30_ (RNA) or 25 pmol unlabeled poly(dA)_30_ (DNA) as competitor. The presence of competitor in the assays is indicated above the panel.

**Figure 6.**
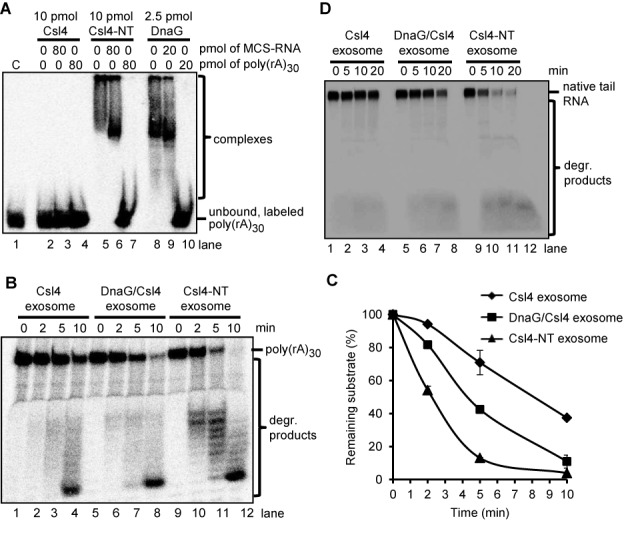
The NTD of DnaG confers strong poly(A) RNA binding capability and poly(A) specificity to the fusion Csl4-NT protein. (**A**) A phosphorimage of EMSA in native 5% PAA gel with the indicated amounts of Csl4, Csl4-NT or DnaG and 25 fmol of labeled poly(rA)_30_. The presence of unlabeled competitors (30-nt MCS-RNA or poly(rA)_30_) in the assays and their amounts is indicated. C, negative control without protein. (**B**) A phosphorimage of a denaturing 16% PAA gel with degradation assays containing 2 pmol of 5′-labeled poly(rA)_30_ and 0.3 pmol of the Csl4 exosome, the DnaG/Csl4-exosome, the Csl4-NT exosome or Csl4-NT. The time of incubation (min) is indicated. The poly(rA)_30_ substrate and the degradation (degr.) products are marked on the right side. (**C**) Graphical representation of the results shown in (B) and of two further independent RNA degradation assays. (**D**) A phosphorimage of a denaturing 16% PAA gel with degradation assays containing 50 fmol internally labeled transcript of 59 nt, which corresponds to a native RNA tail in *Sulfolobus solfataricus* (native tail RNA), and 0.3 pmol of the indicated exosome complexes. The other descriptions are like in (B).

**Figure 7.**
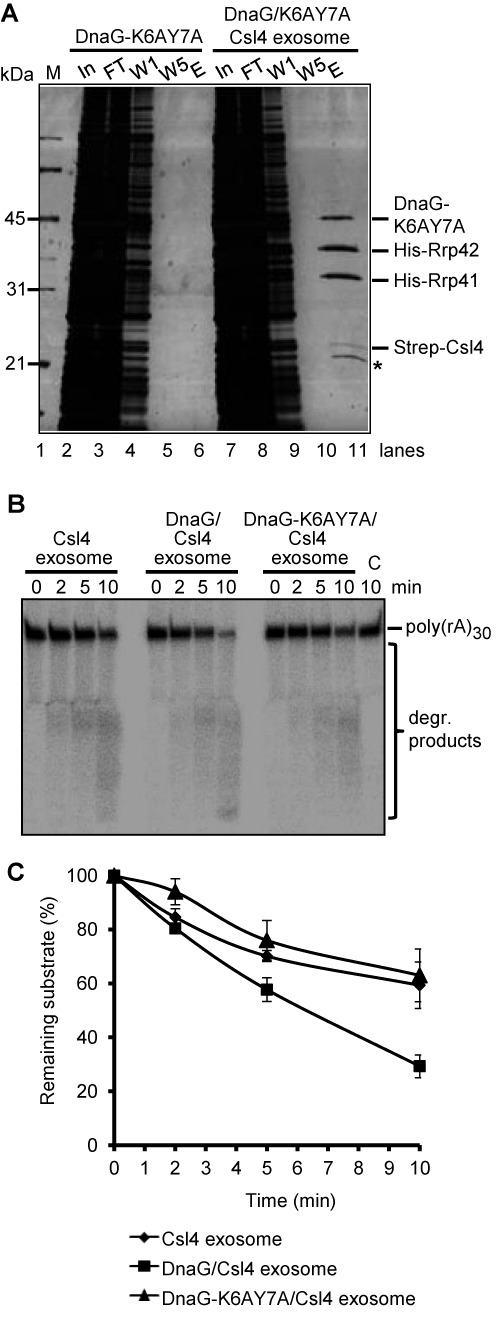
DnaG-K6AY7A binds to the Csl4 exosome but does not influence the degradation of poly(rA)_30_. (**A**) Strep-Csl4 exosome was mixed with DnaG-K6AY7A containing cell-free extract and a pull-down assay with *Strep*-Tactin Sepharose beads was performed (lanes 7–11). As a negative control, the assay was performed with the cell-free extract only (lanes 2–6). M, marker; In, input, the mixture of proteins used; FT, flow-through; W1, W5, the first and the last washing fractions; E, the elution fraction. The protein fractions were analyzed by 12% SDS-PAA gel and silver stained. Relevant proteins are marked on the right side of the panel. The size of marker proteins in kDa is given on the left side. A protein co-purifying with Strep-Csl4 is marked by an asterisk. (**B**) A phosphorimage of a denaturing 16% PAA gel with degradation assays with 8 pmol radioactively labeled poly(rA)_30_ and 0.3 pmol of the Csl4 exosome, DnaG/Csl4 exosome or the DnaG-K6AY7A/Csl4 exosome. The time of incubation (min) is indicated. The poly(rA)_30_ substrate and the degradation (degr.) products are marked on the right side. (**C**) Graphical representation of the results from (B) and two further independent RNA degradation assays.

### The NTD of DnaG is a novel RNA-binding domain

The results of the phylogenetic analysis and the multiple alignments strongly suggest that the NTD of archaeal DnaG has a highly conserved physiological role. Since DnaG from *S. solfataricus* binds poly(rA)_30_ (33), we assumed that the NTD may be involved in binding of RNA. This assumption was strengthened by the similarities between the NTD of archaeal DnaG and other proteins interacting with RNA found by Phyre2 (Supplementary Table S2, Figure 3). There are several invariant amino acid residues in the NTD of archaeal DnaG, among them are the Lys(K)6 and Tyr(Y)7 in DnaG from *S. solfataricus* (Figure 3). Tyr and Lys were reported to play key roles in binding of RNA in several RNA-binding proteins (46,47). Therefore we decided to generate a K6AY7A mutant of DnaG and to test its RNA binding activity by EMSA. The non-tagged, mutated protein was purified and analyzed by circular dichroism spectroscopy in comparison to the recombinant, wild-type DnaG, which carries a His_6_-tag at the C-terminus. No disorder of the secondary structure was detected (Figure 5A and B), allowing us to conclude that the mutated protein is suitable for our analyses.

EMSA assays were performed with the recombinant, wild-type DnaG-His_6_, the K6AY7A mutant and the previously published E175Q mutant of DnaG, which is impaired in the primase activity. As an RNA substrate, poly(rA)_30_, which is easily shifted by DnaG in EMSA was used (33). For comparison, labeled poly(dA)_30_ was used as a DNA substrate. We found that under the applied conditions, poly(dA)_30_ was not bound, while as expected, poly(rA) was strongly bound by wild-type DnaG (compare lane 2 to 6 in Figure 5C). Furthermore, the RNA binding activity of the TOPRIM domain mutant DnaG-E175Q was weaker when compared to wild-type DnaG and RNA binding by the NTD mutant DnaG-K6AY7A was completely abolished (lanes 6 to 8 in Figure 5C). Single mutants DnaG-K6A and DnaG-Y7A were also prepared. They showed very low RNA binding activities (Supplementary Figure S4A and B).

To test whether the E175Q mutant still retained the preference for poly(rA), which is characteristic for the wild-type DnaG, competition assays were performed. Wild-type DnaG and the E175Q mutant were incubated with a mixture of low amount of labeled poly(rA)_30_ and excess of unlabeled poly(rA)_30_ or heteropolymeric MCS-RNA of 30 nt as competitors. Both proteins shifted the labeled poly(rA)_30_ in presence of the MCS-RNA competitor but not in the presence of the poly(rA)_30_ competitor, showing that DnaG-E175Q has poly(rA) preference like wild-type DnaG (Figure 5D). We also performed competition experiments with excess of poly(dA)_30_. Figure 5E shows that 25 pmol of unlabeled poly(dA)_30_ did not have any influence on the strong binding of the labeled RNA by wild-type DnaG, while 2.5 pmol of unlabeled poly(rA)_30_ abolished the binding of the labeled RNA. This shows that DnaG is an RNA-binding rather than DNA-binding protein.

We conclude that the NTD of *S. solfataricus* DnaG is a novel, conserved archaeal RNA binding domain and its K6 and Y7 residues are important for binding of RNA. Furthermore both the NTD and the TOPRIM domain of archaeal DnaG are involved in RNA binding.

### The NTD of DnaG confers strong poly(A) binding capability to a chimeric Csl4-NT protein

Since wild-type DnaG shows a poly(A) preference which is not affected by the E175Q exchange in the TOPRIM domain, we assumed that the NTD is responsible for this preference. To verify this we generated a fusion protein composed of Csl4, which does not bind poly(rA)_30_ strongly and does not show poly(A) preference (32,33), and the NTD of DnaG. As the NTD of Csl4 is the main anchor to the hexameric ring of the exosome (5) and for degradation assays the fusion protein should be capable to interact with the ring, the NTD of DnaG was fused to the C-terminus of Csl4. The fusion, His-tagged protein was named Csl4-NT.

In order to analyze whether the NTD of DnaG influences the RNA binding capability of Csl4, EMSA assays were performed with labeled poly(rA)_30_. The substrate was not shifted by Csl4 (lane 2 in Figure 6A) but was successfully shifted by Csl4-NT and DnaG (lanes 5 and 8 in Figure 6A). Competition with unlabeled MCS-RNA of 30 nt and poly(rA)_30_ in concentrations 8-fold higher than the concentrations of the used proteins revealed that both Csl4-NT and DnaG show poly(rA)-preference (Figure 6A).

DnaG increases the efficiency of degradation of poly(rA)_30_ and A-rich RNA by the Csl4 exosome and by the exosome containing both Csl4 and Rrp4 *in vitro* (33). Here we tested whether the fusion of the NTD of DnaG to Csl4 will have a similar effect. Indeed, the Csl4-NT exosome degraded poly(rA)_30_ faster than the Csl4 exosome and even faster than the Csl4 exosome containing wild-type DnaG (Figure 6B and C). The faster RNA degradation by the Csl4-NT exosome was not due to RNase contamination of the Csl4-NT protein fraction used for reconstitution of the complex, since incubation of poly(rA)_30_ with the Csl4-NT only did not result in degradation (Supplementary Figure S5). Actually, contamination of the degradation assays by spurious RNases originating from *E. coli* were excluded in our assays performed at 60°C (30). Similar results were obtained from degradation assays with an A-rich transcript of 59 nt, which corresponds to a native RNA tail of *S. solfataricius* (Figure 6D). The Csl4-NT containing exosome was the most efficient RNase complex, followed by the Csl4 exosome with DnaG and the Csl4 exosome without DnaG.

In conclusion, the above results show that the NTD of DnaG confers strong binding of poly(rA)_30_ and poly(A)-specificity to the fusion Csl4-NT protein.

### DnaG influences the degradation activity of the Csl4 exosome through binding of RNA

The presence of DnaG stimulates the degradation of A-rich RNA by the Csl4 exosome, most probably because DnaG helps the Csl4 exosome to recruit A-rich substrates (33). We decided to test this assumption experimentally using the DnaG-K6AY7A mutant which cannot bind RNA (Figure 5). First it was necessary to verify that the DnaG-K6AY7A mutant protein still interacts with the exosome. For this a cell-free lysate of the *E. coli* strain producing the DnaG-K6AY7A protein was mixed with reconstituted Strep-Csl4 exosome and purification of Strep-Csl4 containing complexes was performed with *Strep*-Tactin Sepharose beads. Csl4 was detected in the elution fraction together with His_6_-Rrp41, His_6_-Rrp42 and DnaG-K6AY7A (Figure 7A, lane 11). In the control experiment without addition of exosome DnaG-K6AY7A was not present in the elution fraction (Figure 7A, lanes 2 to 6). We conclude that the DnaG-K6AY7A protein was specifically co-purified with the Csl4 exosome.

Next, degradation assays were performed with labeled poly(rA)_30_ and Csl4 exosome, DnaG-containing Csl4 exosome or DnaG-K6AY7A-containing Csl4 exosome. Figure 7B and C show that poly(rA)_30_ is degraded faster in the presence of wild-type DnaG in the protein complex, while there was no significant difference in the degradation of the substrate by the exosome containing DnaG-K6AY7A and the exosome without DnaG. We conclude that the RNA binding capability of DnaG is crucial for its positive influence on RNA degradation by the exosome.

### DnaG enables polynucleotidylation of rRNA by the exosome

**Figure 8.**
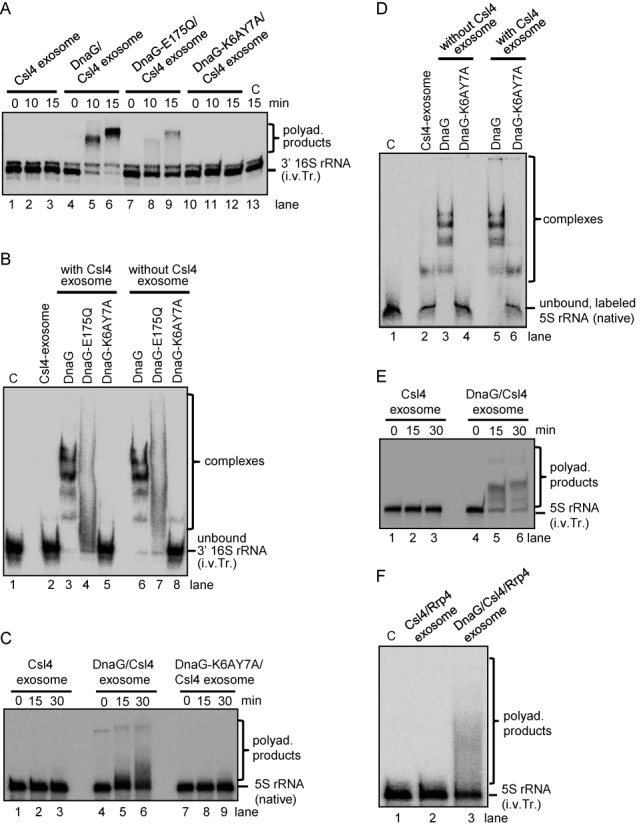
DnaG enables the polyadenylation of rRNA by the exosome. Shown are phosphorimages of assays with an internally labeled *in vitro* transcript (i.v.Tr.) corresponding to the 3′-end of 16S rRNA in *Sulfolobus solfataricus* (3′ 16S rRNA; panels **A** and **B**), 5′-labeled native 5S rRNA (panels **C** and **D**) and internally labeled *in vitro* transcript corresponding to 5S rRNA (panels **E** and **F**). Substrate and reaction products are marked on the right side of the panels. Proteins and protein complexes are indicated above the panels, along with the reaction time (min). C, negative control without protein. (A) Denaturing 10% PAA gel with polyadenylation assays containing 60 fmol of the radioactively labeled substrate and 0.3 pmol of the indicated exosome complexes. (B) EMSA in native 5% PAA gel with 120 fmol of the radioactively labeled transcript and 5 pmol of each of the indicated proteins and protein complexes. (C) Denaturing 10% PAA gel with polyadenylation assays with 70 fmol native 5S rRNA and 0.3 pmol of the indicated exosome complexes. (D) EMSA in native 5% PAA gel with 140 fmol of the radioactively labeled substrate and 5 pmol of each of the indicated proteins and protein complexes. (E) and (F) Denaturing 10% PAA gels with polyadenylation assays. 70 fmol of the 5S rRNA *in vitro* transcript were present in each assay together with 0.3 pmol of the indicated exosome complexes. The incubation time in (F) was 30 min.

Ribosomal RNA is one of the major substrates of the eukaryotic exosome and of bacterial PNPase (20,48). Thus we assumed that in exosome-containing archaea rRNA is also a substrate of the exosome. This assumption is supported by the detection of heteropolymeric A-rich tails, which are most probably synthesized by the exosome, at the 3′-end of 16S rRNA and its fragments in *S. solfataricus* and *Methanopyrus kandleri* (15,34). However, in a previous study a transcript corresponding to the 3′-end of 16S rRNA (3′ 16S rRNA) was not degraded nor polyadenylated *in vitro* by the hexameric Rrp41/Rrp42 ring, the Rrp4 exosome and Csl4 exosome of *S. solfataricus* (30). To test whether DnaG influences the interaction of the exosome with the 3′ 16S rRNA transcript, we performed degradation and polyadenylation tests using the Csl4 exosome with or without DnaG. Interestingly, DnaG enabled polyadenylation of this substrate by the exosome. Even after 15 min of incubation in the presence of rADP, the 3′ 16S rRNA substrate was not polyadenylated by the Csl4 exosome (lanes 1 to 3 in Figure 8A), while after 10 min of incubation with the DnaG-containing Csl4 exosome, the majority of the substrate was prolonged (lanes 4 to 6 in Figure 8A). In contrast DnaG did not enable degradation of the 3′ 16S rRNA transcript by the exosome (Supplementary Figure S6).

To see whether the RNA binding activity of DnaG is important for the positive influence of DnaG on the polyadenylation of the 3′ 16S rRNA transcript by the exosome, DnaG-E175Q and DnaG-K6AY7A were used in the assays instead of wild-type DnaG. Less substrate was polyadenylated by the DnaG-E175Q containing exosome (lanes 7 to 9 in Figure 8A) and the DnaG-K6AY7A containing exosome did not polyadenylate at all (lanes 10 to 12 in Figure 8A). This suggests that binding of the 3′ 16S rRNA transcript by DnaG is necessary for its polyadenylation by the exosome. To test directly whether DnaG binds this transcript, EMSA analyses were performed (Figure 8B). The transcript was completely shifted by the wild-type DnaG and the exosome containing wild-type DnaG, while no comparable shift was observed when the DnaG-K6AY7A protein was used, alone or in the context of the exosome. When DnaG-E175Q was used, the RNA shift was weaker than with the wild-type DnaG, resembling the results obtained with poly(rA)_30_ (compare Figures 5C–8B).

Polyadenylation and EMSA assays were also performed with native 5S rRNA, which was isolated from total RNA of *S. solfataricus* after separation in a 10% urea-polyacrylamide gel and labeled radioactively at the 5′-end. The results were very similar to those obtained with the 16S rRNA-derived transcript: the Csl4 exosome with DnaG polyadenylated the native 5S rRNA, while the exosome without DnaG or with DnaG-K6AY7A did not (Figure 8C). In accordance with this, the native 5S rRNA was strongly shifted by DnaG alone or in combination with the Csl4 exosome in EMSA assays, while no shift was observed when the mutant protein DnaG–K6AY7A was used, and a very weak shift was observed with the Csl4 exosome alone (Figure 8D). Furthermore, we verified that DnaG is also needed for the polyadenylation of *in vitro* transcribed 5S rRNA (Figure 8E). We noticed that although similar amounts of substrate and enzyme were used in the assays shown in Figure 8C and E, the *in vitro* transcript was polyadenylated with higher efficiency than the native 5S rRNA. In contrast to the wild-type DnaG, the double mutant DnaG-K6AY7A and the single mutants did not enable polyadenylation of the 5S rRNA transcript by the exosome (Supplementary Figure S4C).

The above experiments revealed that DnaG enables polyadenylation of rRNA by the Csl4 exosome *in vitro*. However, *in vivo* the exosome contains both Rrp4 and Csl4 (31), and thus we decided to test whether a recombinant exosome containing the two RNA-binding proteins also needs DnaG for polyadenylation of the 5S rRNA transcript. Figure 8F shows that indeed DnaG was necessary for polyadenylation of *in vitro* transcribed 5S rRNA by the exosome containing Rrp4 and Csl4.

### A heteropolymeric tail enhances the degradation of rRNA transcripts by the archaeal exosome

Previously we have shown that in contrast to the non-tailed 3′ 16S rRNA transcript, a tailed variant containing 20 adenine residues at the 3′-end (3′ 16S rRNA-A_20_) can be degraded by the Rrp41/Rrp42 hexamer as well as by Rrp4 exosome and Csl4 exosome (30). Here we tested whether the presence of DnaG influences the degradation of the 3′ 16S rRNA-A_20_ transcript by the exosome containing both Rrp4 and Csl4. We found that DnaG slightly increases the degradation of the tailed transcript by the exosome. Furthermore, distinct intermediate degradation products were detected only when DnaG was present in the exosome (Supplementary Figure S7).

**Figure 9.**
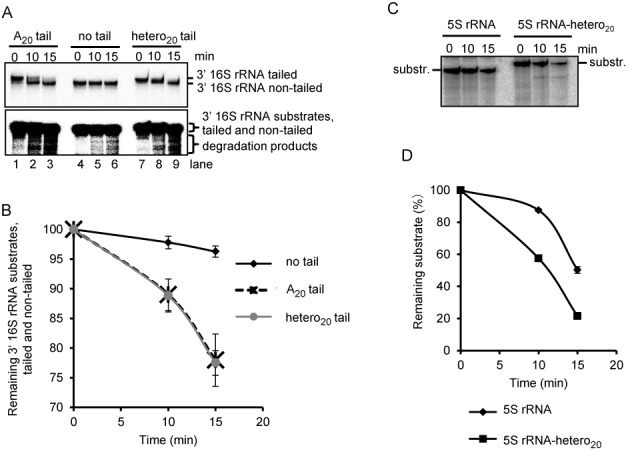
A heteropolymeric tail increases the degradation of rRNA derived transcripts by the exosome. Shown are phosphorimages of degradation assays with 0.3 pmol of the DnaG/Csl4/Rrp4 and 60 fmol of each of the indicated, internally labeled *in vitro* transcripts. (**A**) Denaturing 16% PAA gel with assays containing 3′ 16S rRNA, 3′ 16S rRNA-A_20_ (with a poly(A) tail of 20 nt) or 3′ 16S rRNA-hetero_20_ (with an A-rich, heteropolymeric tail of 20 nt), as indicated above the panels. The reaction time at 60°C is also indicated. Upper panel, short time exposure allowing the differentiation between tailed and non-tailed transcripts. Lower panel, long time exposure allowing the detection of degradation products. Substrates and degradation products are marked on the right side. (**B**) Graphical representation of the results from (A) and two further independent RNA degradation assays. (**C**) Denaturing 16% PAA gel with assays containing 5S rRNA or 5S rRNA-hetero_20_ transcripts. For conditions and descriptions, see (A). (**D**) Graphical representation of the results from (C) and two further independent RNA degradation assays.

Next we analyzed the influence of a heteropylmeric tail on the degradation of 3′ 16S rRNA by the exosome containing Rrp4, Csl4 and DnaG. We compared the degradation of the non-tailed transcript to that of its tailed derivatives 3′ 16S rRNA-A_20_ and 3′ 16S rRNA-hetero_20_ containing a poly(A) tail or a heteroplymeric tail of 20 nt, respectively. The sequence of the heteropolymeric tail corresponds to a tail sequence previously detected in *S. solfataricus* (15). We observed that the degradation of the tails restoring the non-tailed transcript was faster than degradation of the body of the transcript (compare the two panels of different exposition in Figure 9A). Furthermore, considering degradation products shorter than 3′ 16S rRNA, we found that both tailed transcripts are degraded faster than the non-tailed one and that both tails equally enhance the degradation (Figure 9A and B). We also tested whether the heteropolymeric tail leads to faster degradation of the 5S rRNA transcript by the exosome. As expected, the tailed variant was degraded faster (Figure 9C and D).

## DISCUSSION

Our phylogenetic analysis suggests that archaeal DnaG is an ancient protein predating the origin of the archaeal kingdom, since the five archaeal phyla Euryarchaeota, Crenarchaeota, Nanoarchaota, Korarchaeota and Thaumarchaeota were delineated in a very similar way in the DnaG and 16S rRNA phylogenetic trees (Figure 2 and Supplementary Figure S1). However, our analysis also shows that the presence or absence of exosome had an influence on the evolution of DnaG in Archaea. Previously archaeal DnaG sequences were used for phylogenetic analysis of methanogenic consortia leading to very similar results when compared to 16S rRNA-based analysis (49). Probably this was due to the phylogenetic homogeneity of the studied archaeal group, in which no differences in respect of the exosome content are expected. We found substantial differences in the subtree of Euryarchaeota comprising archaea with and without exosome. Thus, despite its high conservation, archaeal DnaG is not suitable as a phylogenetic marker.

Protein–protein interaction studies with truncated DnaG proteins revealed that the NTD is not essential for the interaction with the exosome and that the CTD is important for this interaction (Figure 4). An involvement of the CTD in binding of DnaG to the exosome is also supported by the sequence comparisons shown in Figure 3, Supplementary Figures S2 and S3, since higher conservation of the CTD was found in exosome-containing than in exosome-less archaea. Not only DnaG-ΔCT but also DnaG-ΔNT is impaired in its interaction with the Csl4-exosome (compare Figures 4D–7A). Thus, the integrity of DnaG is important for a strong binding to the exosome. Most probably the conformation of the CTD is changed in the truncated DnaG-ΔNT protein preventing efficient binding to the protein complex. Alternatively or in addition, each domain may contribute to the interaction with the exosome. It is known that the TOPRIM domain of the RecR protein from *E. coli* is responsible for the interaction with other protein partners and with DNA (50). The overall spatial structure of the archaeal DnaG-containing exosome is still not known. In exosome-less archaea the CTD of DnaG may contribute to the integrity of the protein and/or to the interaction with other proteins.

Our data clearly show that the NTD is a novel, conserved archaeal RNA-binding domain, which is essential for the interaction of *S. solfataricus* DnaG with RNA (Figure 5). The experiments with the chimeric Csl4-NT protein revealed that the NTD of DnaG is a separate RNA binding domain with poly(A)-preference, which can exert this function in the context of different proteins (Figure 6). We also show that the NTD is needed for strong binding of 5S rRNA and rRNA-derived transcripts (Figure 8B and D). Thus, despite its poly(A)-preference, this protein domain is a general RNA-binding domain necessary for interaction of archaeal DnaG with heteropolymeric substrates. Interestingly, we found that the TOPRIM domain is also involved in the interaction of DnaG with RNA. Notably, the conserved residue E175 in the TOPRIM domain, which is crucial for the primase activity of the protein (35), is important for strong RNA binding by DnaG (Figures 5 and 8). These results strongly suggest that the NTD and TOPRIM domains cooperate in binding of RNA substrates. Cooperation of multiple RNA binding domains, each with a weak affinity for RNA, is known to result in a strong RNA binding by other proteins involved in RNA metabolism like Lin28, a major regulator in mammalian cells, and the eukaryotic mRNA export factor Tip-associated protein; Tip is a tyrosine kinase-interacting protein (TAP) (51). The TOPRIM domain is characteristic for bacterial type primases, topoisomerases, OLD family nucleases and RecR proteins, altogether proteins involved in interactions with DNA (26). However, archaeal DnaG is not the only protein with a TOPRIM domain which binds RNA. A prominent example is ribonuclease (RNase) M5 from *Bacillus subtilis*, in which a TOPRIM domain contains the active site. Both the TOPRIM and the CTD of RNase M5 are important for binding of RNA (52).

Binding of RNA by DnaG is important for the observed faster degradation of poly(rA)_30_ (Figure 7) and is a prerequisite for the polyadenylation of rRNA and rRNA-derived transcripts by the DnaG-containing exosome *in vitro* (Figure 8). Therefore we propose that in archaea harboring exosome DnaG not only participates in the efficient interaction of A-rich RNA with the exosome (33), but is also responsible for the polynucleotidylation of rRNA. It is assumed that the heteropolymeric, A-rich RNA tails in exosome-containing archaea have destabilizing function (15,17) like the short poly(A) tails in enterobacteria and eukarya (18,19,53,54). Our data, showing that a heteropolymeric tail leads to faster degradation of rRNA transcripts by the exosome *in vitro*, are in agreement with this assumption. The destabilizing effect of the heteropolymeric tail was comparable to the effect of a poly(A) tail of the same length (Figure 9B). Similarly, both a heteropolymeric tail and a poly(A) tail equally enhanced the degradation of structured RNA by the bacterial degradosome *in vitro* (54). Analyses of the nucleotide composition of bacterial and archaeal heteropolymeric RNA tails suggested that the tails do not have potential to form strong secondary structures (32,55). Together, these data support the view that prokaryotic, heteropolymeric tails function as single stranded regions enabling fast initial interaction of RNA substrates with 3′–5′ exoribonuceases. Although *in vivo* data demonstrating the destabilizing role of heteropolymeric tails in prokaryotes are still missing (55), we suggest that DnaG plays an important role in degradation of rRNA in exosome-containing archaea. This suggestion is based on the data shown in Figures 8 and 9. Degradation of rRNA in the course of the quality control during ribosome biogenesis or as adaptation to changing environmental conditions is of pivotal importance for the cell (18,20,56).

The *in vitro* polyadenylation of the native 5S rRNA was less efficient than the polyadenylation of the *in vitro* transcribed 5S rRNA, although comparable substrate amounts were used in the assays (compare lanes 4 to 6 in Figure 8C and 8E). This can be explained by the failure of some *in vitro* transcripts to adopt the native rRNA structure. Additionally, missing RNA modifications can lead to lower stability RNA structures (57) and this can lead to higher accessibility of the 3′-end of the transcript for tailing by the exosome. Furthermore T7 polymerase adds a non-templated nucleotide at the 3′-end of *in* *vitro* transcripts (58), which may facilitate addition of poly(A) by the DnaG containing exosome. We also observed that the 5S rRNA transcript is polyadenylated much faster by the DnaG/Csl4 exosome than the DnaG/Csl4/Rrp4 exosome (compare Figure 8E, lane 6, to Figure 8F, lane 3). Most probably this is due to the lower amount of DnaG in the Rrp4 containing exosome (Figure 1B)). *In vivo* exosomal complexes with different stoichiometric amounts of Rrp4 and DnaG/Csl4 are present (31). Probably archaeal exosomes of different compositions exhibit different functions, and it is possible that the exosomal complexes with higher relative amounts of DnaG are responsible for tailing of stable RNA.

Our results characterizing *S. solfataricus* DnaG as an RNA binding subunit of the archaeal exosome do not necessarily exclude a function of DnaG as a primase in the cell (35,36). It is possible that archaeal DnaG is a moonlighting protein like some other proteins with more than one function in prokaryotes and eukaryotes (59). However there are several reasons to believe that it is rather involved in RNA metabolism than in replication (60,61). The strong *in vivo* interaction with the exosome in several archaea was already mentioned in the introduction (8,12,13,31). *In vitro* this interaction leads to a clear and strong effect of DnaG on the polynucleotidylation of native rRNA and rRNA-derived transcripts by the exosome (Figure 8). In comparison, the documented interaction between *S. solfataricus* DnaG and the MCM helicase is weak (36). Importantly, this interaction does not influence the priming activity of DnaG and specifically inhibits the helicase activity of MCM (36). This is in contrast to the enhanced helicase activity of DnaB and the priming activity of DnaG upon interaction between bacterial DnaG and DnaB (62–64). Additionally, the Phyre2 analysis of the NTD and CTD domains of DnaG from the exosome-containing *S. solfataricus* and the exosome-less *M. jannaschii* revealed similarities between archaeal DnaG and bacterial and eukaryotic proteins involved in RNA metabolism (Supplementary Table S2), but no connection to the archaeal replication network was found. Together with the high, exosome-independent conservation of the NTD in archaea, the strong affinity of this NTD for RNA but not DNA, and the involvement of the TOPRIM domain in RNA binding, this implicates that DnaG functions as an RNA binding protein even in archaea lacking an exosome. In exosome-less archaea DnaG may play a role in the process of RNA degradation together with archaeal homologs of the bacterial RNases R and J, or of the eukaryotic cleavage and polyadenylation specificity factor (34,65–68). According to our data, DnaG is most probably involved in tailing and degradation of stable RNAs in exosome-containing archaea.

## SUPPLEMENTARY DATA

Supplementary Data are available at NAR Online.

SUPPLEMENTARY DATA
