# Supplementary material for: Archaeal DnaG contains a conserved N-terminal RNA-binding domain and enables tailing of rRNA by the exosome
Source: Nucleic Acids Res. 2014 Oct 17;42(20):12691–706. doi: 10.1093/nar/gku969 (PMC4227792; doi:10.1093/nar/gku969)
Supplement: SUPPLEMENTARY DATA [file supp_42_20_12691__index.html]

Archaeal DnaG contains a conserved N-terminal RNA-binding domain and enables tailing of rRNA by the exosome — Archaeal DnaG contains a conserved N-terminal RNA-binding domain and enables tailing of rRNA by the exosome — SUPPLEMENTARY DATA 

# Archaeal DnaG contains a conserved N-terminal RNA-binding domain and enables tailing of rRNA by the exosome

## SUPPLEMENTARY DATA

**Files in this Data Supplement:**

- SUPPLEMENTARY DATA
