# Supplementary material for: Archaeal DnaG contains a conserved N-terminal RNA-binding domain and enables tailing of rRNA by the exosome
Source: Nucleic Acids Res. 2014 Oct 17;42(20):12691–706. doi: 10.1093/nar/gku969 (PMC4227792; doi:10.1093/nar/gku969)
Supplement: SUPPLEMENTARY DATA [file supp_gku969_nar-01844-r-2014-File011.pdf]

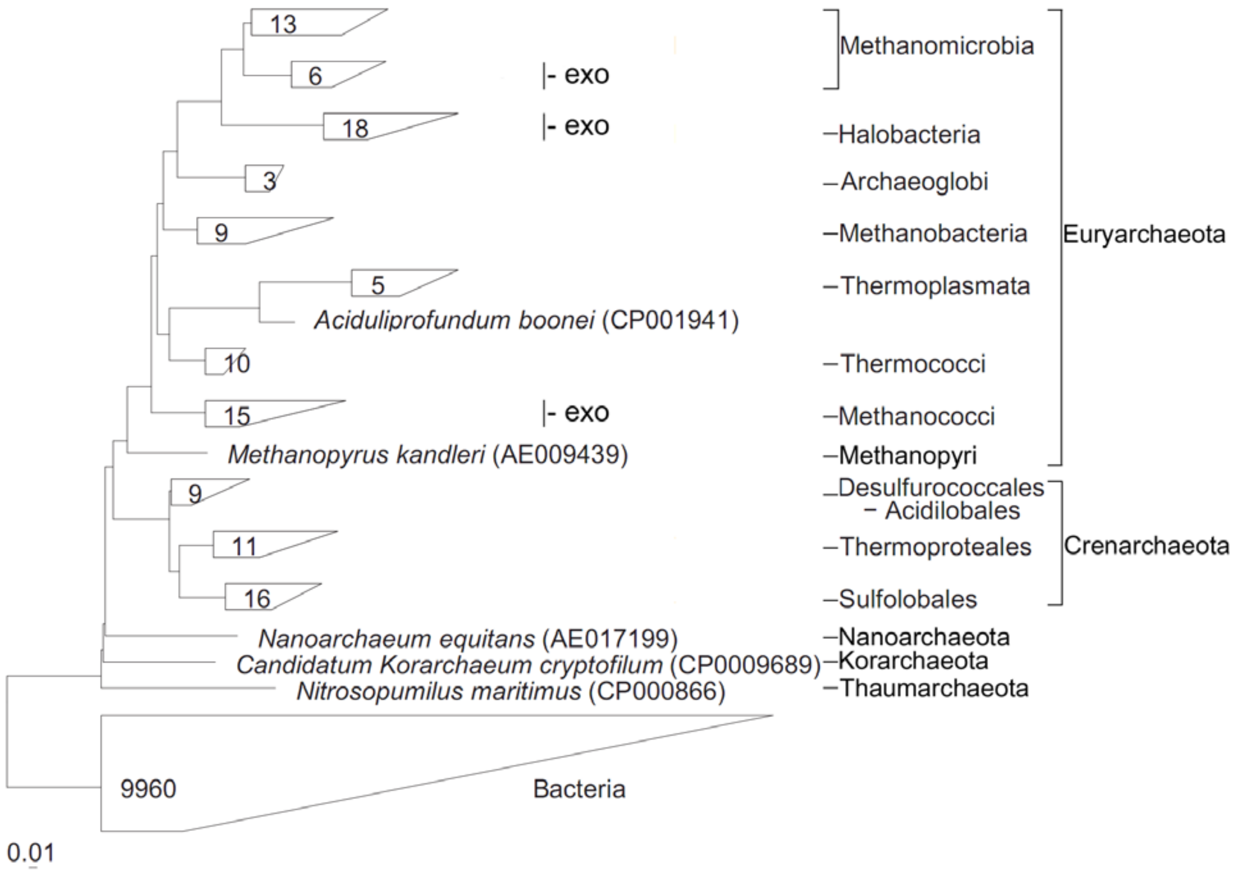

Figure S1. Phylogenetic analysis of 16S rRNA genes in archaea. Phylogenetic tree of 16S rRNA genes in genome-sequenced Archaea. Sequences of 16S rRNA genes were gained from the LTP database and NCBI (http://www.ncbi.nlm.nih.gov/) and aligned using SILVA Incremental Aligner (SINA) version v1.2.1167. The maximum-likelihood phylogenetic tree was obtained in ARB release 5.265 using the “All-Species Living Tree" Project (LTP66) ARB database release LTPs111 (February 2013) based on the alignment. The number of the genome-sequences per clade is indicated. All archaea have exosome, with the exception of Methanococci, Halobacteria and some Methanomicrobia (-exo, archaea without exosome).

The tree was generated with the kind help of Dr. Stephanie Glaeser (Institute for Applied Microbiology, University of Giessen, Germany).

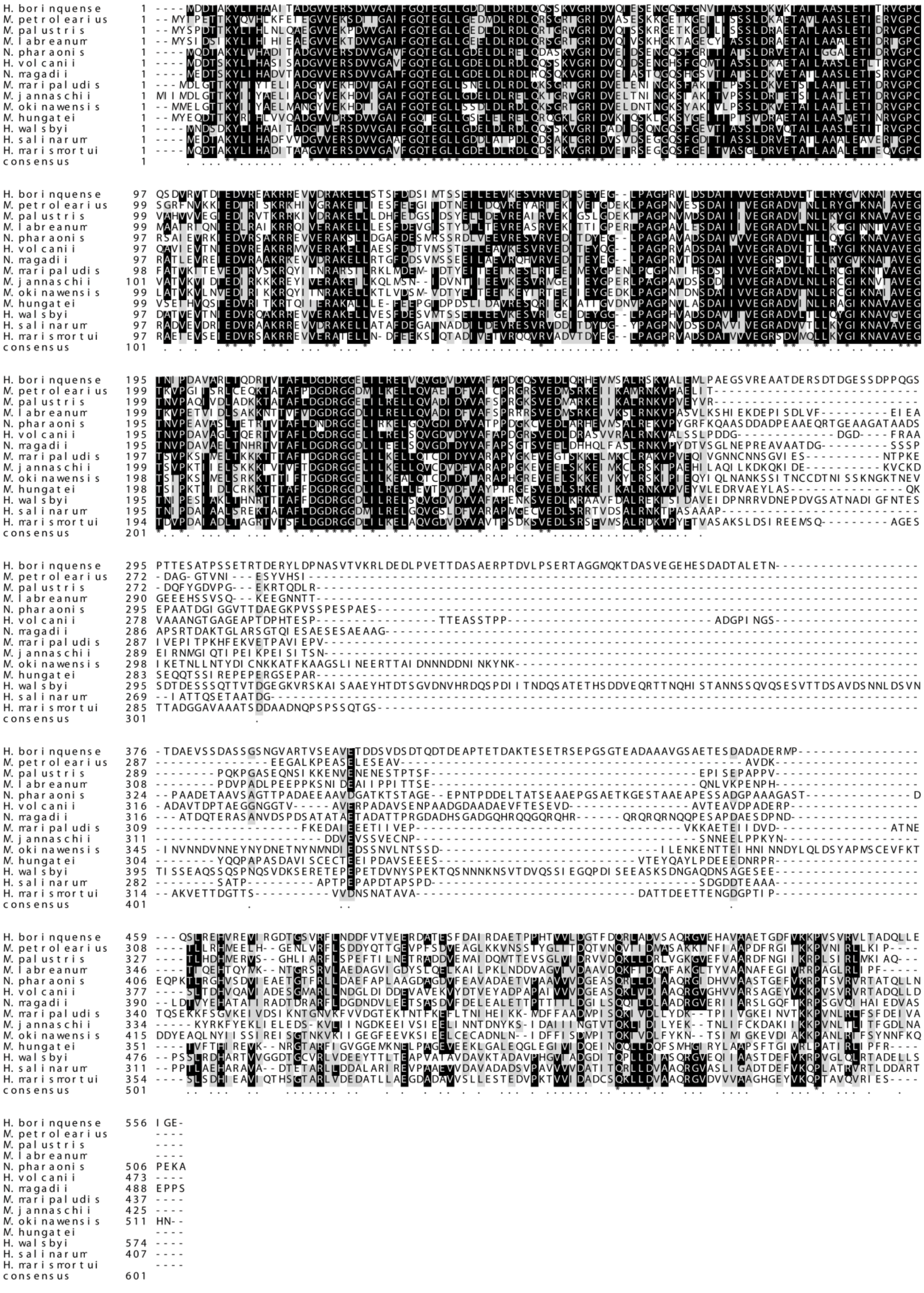

Figure S2. Alignment of DnaG from archaea which have no exosome. Three invariant residues (Q387, D391 and P411 in *M. jannaschii*) are present in the last 100 amino acid residues of the C-terminal domain of DnaG. All exosome-less archaea belong to Euryarchaeota**.**

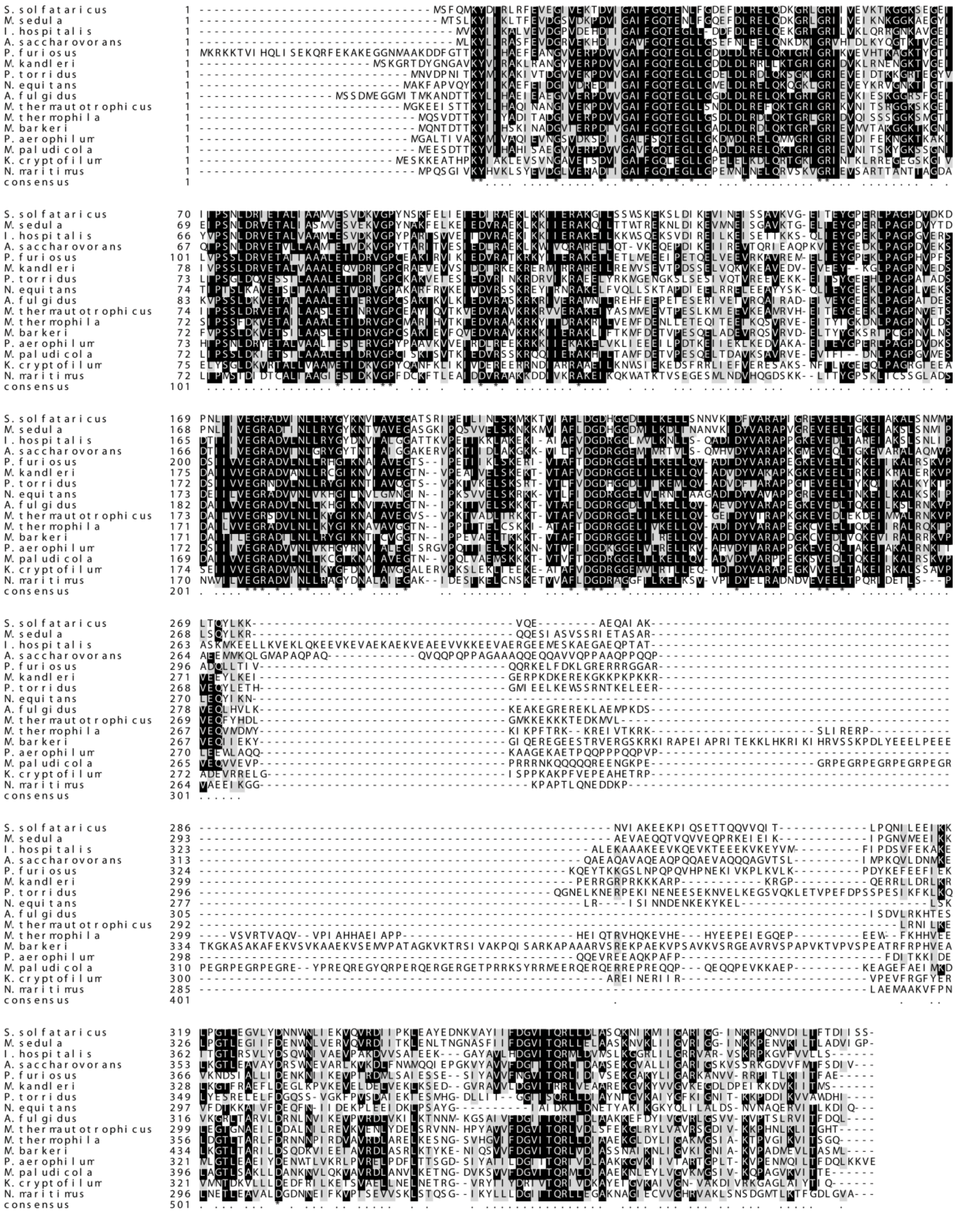

Figure S3. Alignment of DnaG from archaea which have exosome. Representatives of Euryarchaeota, Crenarchaeota, Nanoarchaota, Korarchaeota and Thaumarchaeota were included in the analysis. In the last 100 aa of the C-terminal domain, an invariant aspartate residue (D329 in *S. solfataricus*) and a cluster of conserved residues (F360 to D367 in *S. solfataricus*) are present.

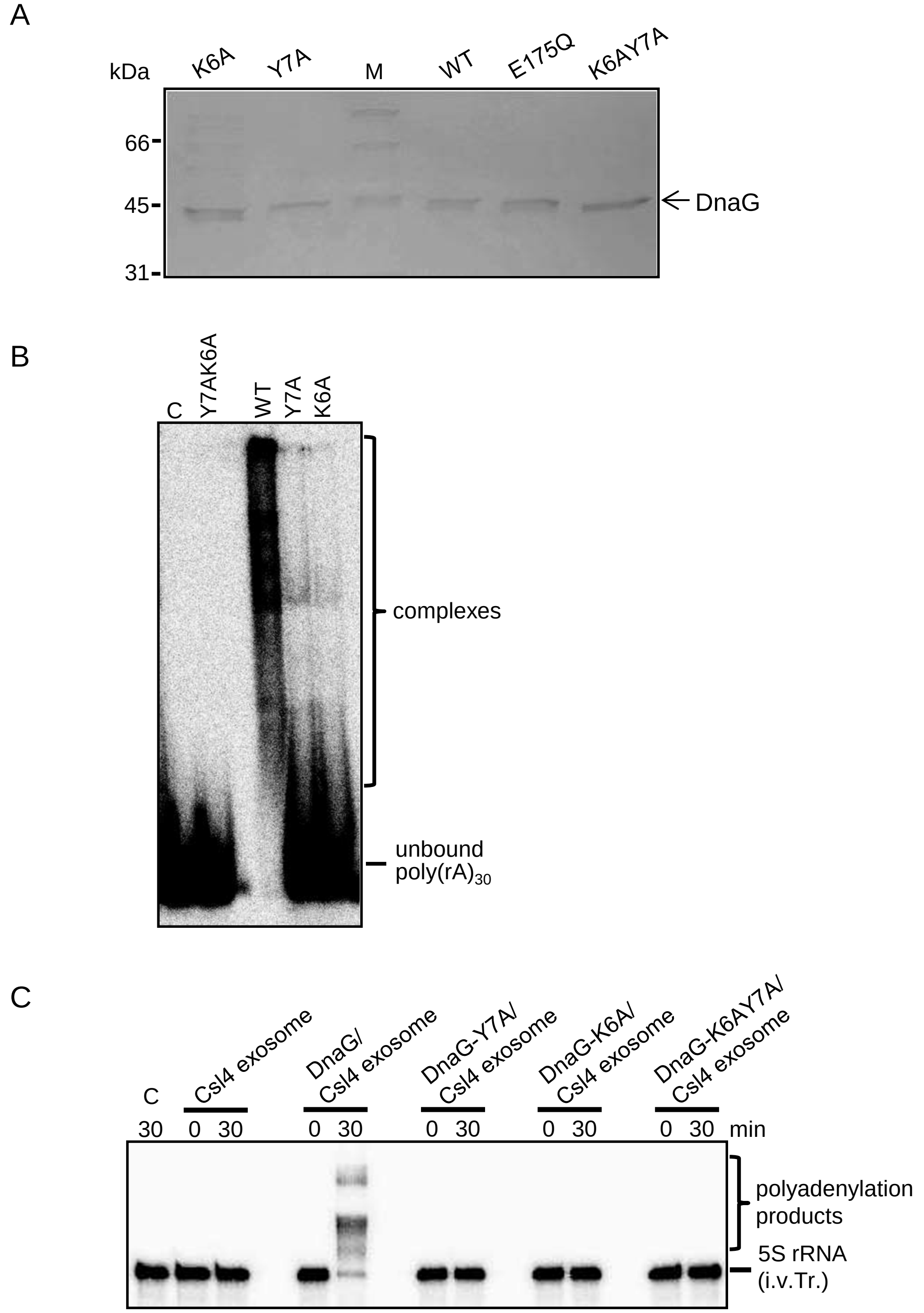

Figure S4. Purification and analysis of the single mutant proteins DnaG-K6A and DnaG-Y7A. A) SDS-PAGE analyis of the DnaG proteins used in this study as indicated above the panel. WT, wild type, recombinant DnaG-His$_6$ protein. The gel was stained with Coommassie. M, protein marker, migration of the marker proteins in kDa is indicated at the left side. B) EMSA of the DnaG proteins indicated above the panel with poly(rA)$_{30}$. Concentrations of proteins and RNA are like in Fig. 5. C) Polyadenylation assays with the 5S rRNA *in vitro* transcript and the exosomal complexes indicated above the panel. Incibation time at 60° C is also indicated. C, negative control without protein. Concentrations of substrate and enzyme are specified in Fig. 8.

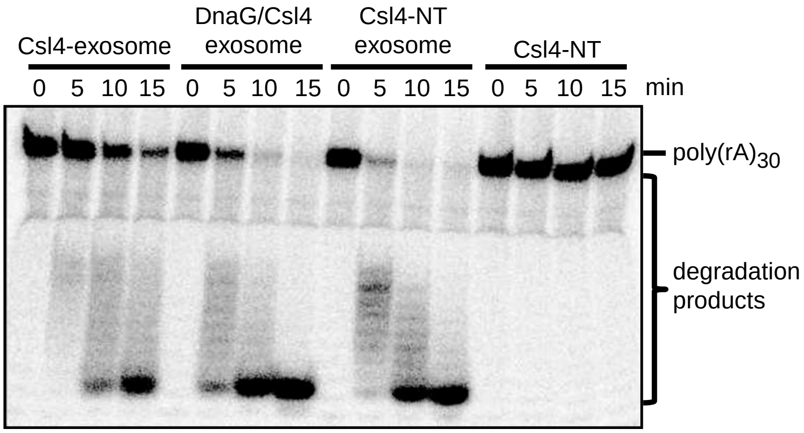

Figure S5. The fusion protein Csl4-NT increases the degradation of poly(rA)$_{30}$ by the exosome, while the Csl4-NT fraction does not exhibit RNase activity. Degradation assays were performed at 60°C for the indicated time. Concentrations of substrate and enzyme are specified in Fig. 6.

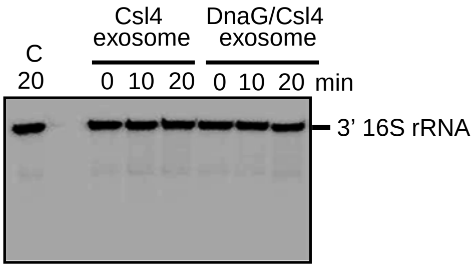

Figure S6. DnaG does not promote degradation of a transcript corresponding to the 3'-end of 16S rRNA by the exosome. Degradation assays were performed at 60°C for the indicated time. Concentrations of substrate and enzyme are specified in Fig. 8.

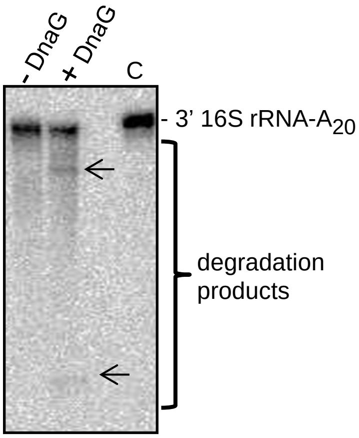

Figure S7. DnaG increases the degradation of the 3’ 16S rRNA transcript carying a poly(A) tail of 20 nt (3’ 16S rRNA-A_{20}) by the exosome containing. Phosphorimage of a denaturing 16% PAA gel with degradation assays containing 1.000 c. p. m. internally labeled 16S rRNA-A_{20} transcript and 0.3 pmol of the Csl4/Rrp4 exosome with or without DnaG as indicated above the panel. The assays were performed at 60°C for 30 min. Substrate (3’ 16S rRNA-A_{20}**)** and degradation products are marked on the right side. Abundant intermediate degradation products are marked with arrows. C, negative control without protein.

**Primers for DnaG-ΔCT cloning**	
DnaG-ΔCT-fwd	5'-catatg gtgagcttccaaatgaaatatgatata-3'
DnaG-ΔCT-rev	5'-ctcgag gccaatcggtgctcttgctac-3'
**Primers for DnaG-ΔNT cloning**	
DnaG-ΔNT-fwd	5'- catatg gaaagattacccgcaggcccagac-3'
DnaG-ΔNT-rev	5'-ctcgag agaagaaataatatcggtaaatgtc-3'
**Primers for Csl4-NT cloning**	
Csl4-NT-fwd	5'-catatg gtgagaactcaaggcgaatt-3'
Csl4-NT-mid-fwd	5'- tcatatttcatttggaagctcacatttccacctttcttcacca -3'
Csl4-NT-mid-rev	5'- tggtgaagaaaggtggaaatgtgagcttccaaatgaaatatga -3'
Csl4-NT-rev	5'-ggatcc tggtccatattctgttatttcc-3'
**Primers for DnaG-K6AY7A cloning**	
DnaG-K6AY7A-fwd	5'-ccatgg atgagcttccaaatggcagctgatataaggttaa-3'
DnaG-rev	5'-ctcgag ctaagaagaaataatatcggtaaatg-3'
**Primers for DnaG-K6A cloning**	
DnaG-K6A-fwd	5'-ccatgg atgagcttccaaatggcatatgatataaggttaa-3'
DnaG-rev	5'-ctcgag ctaagaagaaataatatcggtaaatg-3'
**Primers for DnaG-Y7A cloning**	
DnaG-Y7A-fwd	5'-ccatgg atgagcttccaaatgaaagctgatataaggttaa-3'
DnaG-rev	5'-ctcgag ctaagaagaaataatatcggtaaatg-3'
**Primers for 5S rDNA amplification**	
5S rRNA-fwd	5'-*taatacgactcactataggg*gcccacccggtcacagtgag-3'
5S rRNA-rew	5'-aaaacccatcccagcttagtgggg-3'
5S rRNA-A_{20}-rew	5'-ttttttttttttttttttttaaaacccatcccagcttagtgggg-3'
5S rRNA-$hetero_{20}$-rew	5'-tctttattttatccccctttaaaacccatcccagcttagtgggg-3'
**Primers for 16S rDNA amplification**	
16S rRNA-fwd	5′-g*taatacgactcactataggg*tgaatacgtccctgctcc-3′
16S rRNA-rew	5′-ggaggtgatccagccgcag-3′
16S rRNA-A_{20}-rew	5'-ttttttttttttttttttttggaggtgatccagccgcag-3'
16S rRNA-$hetero_{20}$-rew	5'-tctttattttatccccctttggaggtgatccagccgcag-3'

Table S1. Oligonucleotides used in this study.

Sequences corresponding to restriction sites are underlined .
The T7 promoter sequence is in italics.

S. solfataricus DnaG		
**NTD**		
Region	Confidence*	Highest scoring templates information
AA12-56	50.5%	structure of RNA binding domain (residues 404-479) of *Bacillus subtilis* ATP-dependent RNA helicase YxiN protein
AA87-123	65.9%	structure of a mammalian ribosomal 60S subunit (60S ribosomal protein L32) within an 80S complex
**CTD**		
Region	Confidence*	Highest scoring templates information
AA353-379	57.9%	crystal structure of the human transcription elongation factor DSIF hSpt4 /hSpt5 (176-273)
AA338-380	42.72%	Superfamily: NAD(P)-binding Rossmann-fold domains<br>Family: CoA-binding domain

M. jannaschii DnaG		
**NTD**		
Region	Confidence*	Highest scoring templates information
AA16-60	47.6%	structure of RNA binding domain (residues 404-479) of *Bacillus subtilis* ATP-dependent RNA helicase YxiN protein
AA91-127	66.6%	structure of a mammalian ribosomal 60S subunit (60S ribosomal protein L32) within an 80S complex
**CTD**		
Region	Confidence*	Highest scoring templates information
AA373-407	31.3%	crystal structure of the human transcription elongation factor DSIF hSpt4 /hSpt5 (176-273)

*Confidence indicates the probability of being homologous according to Phyre2 (http://www.sbg.bio.ic.ac.uk/phyre2/html/page.cgi?id=index).

Table S2. Similarity of the NTD and CTD of archaeal DnaG to other proteins detected by Phyre2 (Protein Homology/analogY Recognition Engine V 2.0).
